# Proprioceptive Localization of the Hand Changes When Skin Stretch around the Elbow Is Manipulated

**DOI:** 10.3389/fpsyg.2016.01620

**Published:** 2016-10-21

**Authors:** Irene A. Kuling, Eli Brenner, Jeroen B. J. Smeets

**Affiliations:** Department of Human Movement Sciences, MOVE Research Institute Amsterdam, Vrije Universiteit AmsterdamAmsterdam, Netherlands

**Keywords:** skin stretch, proprioception, hand localization

## Abstract

Cutaneous information has been shown to influence proprioceptive position sense when subjects had to judge or match the posture of their limbs. In the present study, we tested whether cutaneous information also affects proprioceptive localization of the hand when moving it to a target. In an explorative study, we manipulated the skin stretch around the elbow by attaching elastic sports tape to one side of the arm. Subjects were asked to move the unseen manipulated arm to visually presented targets. We found that the tape induced a significant shift of the end-points of these hand movements. Surprisingly, this shift corresponded with an increase in elbow extension, irrespective of the side of the arm that was taped. A control experiment showed that this cannot be explained by how the skin stretches, because the skin near the elbow stretches to a similar extent on the inside and outside of the arm when the elbow angle increases and decreases, respectively. A second control experiment reproduced and extended the results of the main experiment for tape on the inside of the arm, and showed that the asymmetry was not just a consequence of the tape originally being applied slightly differently to the outside of the arm. However, the way in which the tape was applied does appear to matter, because applying the tape in the same way to the outside of the arm as to the inside of the arm influenced different subjects quite differently, suggesting that the relationship between skin stretch and sensed limb posture is quite complex. We conclude that the way the skin is stretched during a goal-directed movement provides information that helps guide the hand toward the target.

## Introduction

Many daily-life goal-directed movements can be regarded as visuo-proprioceptive tasks: They involve combining visual information (about a target) with visual information and proprioception (of an effector). For example, when playing tennis, visual information about the position of the ball is combined with visual and proprioceptive information about the position of the hand (and the tennis racket) to plan an attacking movement. Proprioception is based on a combination of afferent information from cutaneous, muscle spindle and joint receptors, and knowledge of the motor command (McCloskey, 1978; Gandevia et al., 2006; Proske and Gandevia, 2012). The combination of multiple information sources is important for understanding proprioception. For example, no distortions on perceived hand and arm positions were found in visuo-proprioceptive tasks in which the arm was exposed to external forces (Cordo and Flanders, 1989; Kuling et al., 2013, 2015), which suggest that the sources are combined in a way that separates judged positions from the exerted force, which is probably beneficial if one wants to be able to move to specified positions while holding objects of different weights.

Although muscle spindle information is generally assumed to be the most relevant source of proprioceptive information, the role of cutaneous receptors has also been studied extensively in tasks directly related to the configuration of the limb. In the last decades many experiments on finger configurations and movements have shown that cutaneous information influences proprioceptive position sense (Edin and Abbs, 1991; Edin and Johansson, 1995; Collins and Prochazka, 1996; Collins et al., 2000; Weerakkody et al., 2007). The importance of cutaneous information for the perception of finger configuration may arise from the fact that for a finger, the same muscle lengths may correspond with multiple finger configurations (because finger muscles span several joints), so cutaneous information is necessary to determine the individual joint angles (Collins et al., 2000). However, recent studies show that also the perceived angle of the elbow (Collins et al., 2005), ankle (Aimonetti et al., 2007), and knee (Edin, 2001; Collins et al., 2005) can be influenced by cutaneous information, despite the fact that for these joints, the angle can be determined on the basis of muscle length information (from muscle spindles) alone.

In their study, Collins et al. (2005) asked subjects to continuously match the joint angle of the index finger, arm, or leg with the angle of the same joint of the limb on the other side of the body. In the experiment with the arm, the skin stretch around the elbow joint was manipulated by pulling strings that were attached to the skin with a piece of tape. Weak skin stretch evoked a significant illusion in the perceived elbow flexion for 4 out of 10 subjects and stronger stretch evoked the illusion for 5 out of 10 subjects. Overall, the skin stretch induced a significant effect in perceived elbow angle, both with and without additional vibration of the muscle spindle receptors that discharge during joint flexion.

In most experiments that investigated the influence of cutaneous information on proprioception, the cutaneous manipulation was done using anesthesia (Edin and Johansson, 1995) or vibrations (Weerakkody et al., 2007), or combining vibrations with elastic wires attached to the skin (Collins and Prochazka, 1996; Collins et al., 2000, 2005). In the above-mentioned studies subjects had to match the configuration of the manipulated limb with the corresponding limb on the other side of the body (Edin and Johansson, 1995; Collins and Prochazka, 1996; Collins et al., 2000, 2005), or to verbally judge the direction of the illusory joint movements (Weerakkody et al., 2007). In these tasks, subjects directly compared two postures proprioceptively (matching task) or indicated a direction of joint movement. It is interesting to also look at the effect of manipulation of cutaneous information in active reaching, in which both proprioception and the efference copy of the motor command play a role. In previous research involving proprioception and active reaching, we showed that various manipulations with force fields did not influence the reached end positions of the hand, suggesting that subjects could effectively correct for external forces (Kuling et al., 2013, 2015). If this was due to the availability of reliable cutaneous information, manipulating cutaneous information from the arm should influence the proprioceptive position sense of the hand during active reaching. We examined whether it does so.

For the cutaneous manipulation, we used elastic therapeutic tape (Cure Tape®), because it can produce skin stretch in an easy, non-invasive manner. Cure Tape® is an elastic cotton tape and is comparable with Kinesio Tex® Tape (e.g., Kase et al., 2013). Elastic tape is used in rehabilitation and after injury for its claimed therapeutic benefits. The pressure and the stretching effect provoked by elastic tape application on the skin is believed to stimulate cutaneous mechanoreceptors (Grigg, 1994). Whether elastic tape affects proprioceptive accuracy is unclear. Halseth et al. (2004) did not find significant changes in a proprioceptive matching task with tape around the knee, while Callaghan (1997) found improvements for subjects with poor proprioceptive ability. The use of the tape was also found to reduce the timing variability in rhythmic hand movements (Bravi et al., 2014). Our main reason for using the tape is that it is cheap, simple and non-invasive, and allows us to flexibly apply strain to various regions of the skin.

In the present, explorative study, we tested whether manipulating skin stretch around the elbow influences the proprioceptive position sense of the hand in a visuo-proprioceptive matching task that involves active reaching. If people switch between sources of information when they notice that one is not reliable, as might have occurred for our previous force field manipulations (Kuling et al., 2013), and as might happen here if they feel the tape pulling on their arm, manipulating skin stretch might only be effective if we combine cutaneous manipulation with a disturbing force and vice versa. We therefore also examined whether disrupting both the cutaneous and muscle information leads to larger effects on visuo-proprioceptive matching than either alone. In the main experiment, the effect of tape on proprioceptive position sense of the hand was investigated in a visuo-proprioceptive matching task with and without an external force on the hand. In two control experiments we examined the actual skin stretch patterns of the arm during elbow flexion and extension and varied the details of how the tape was applied.

## Methods

### Subjects

Sixteen subjects (three left-handed, two men, 18–22 years of age) volunteered to take part in the main experiment. Eleven right-handed subjects (one male, 20–28 years of age) volunteered to take part in control experiment 1. Thirteen subjects (all right-handed, all female, 19–28 years of age) volunteered to take part in control experiment 2. All subjects had (corrected-to-) normal vision and were naive about the purpose of the experiment.

All subjects gave written informed consent prior to participation and the experimental procedures were in accordance with the Declaration of Helsinki. The experiment is part of an ongoing research program that has been approved by the ethics committee of the Department of Human Movement Sciences of Vrije Universiteit Amsterdam.

### Stimulus and apparatus

**Figure 1:**
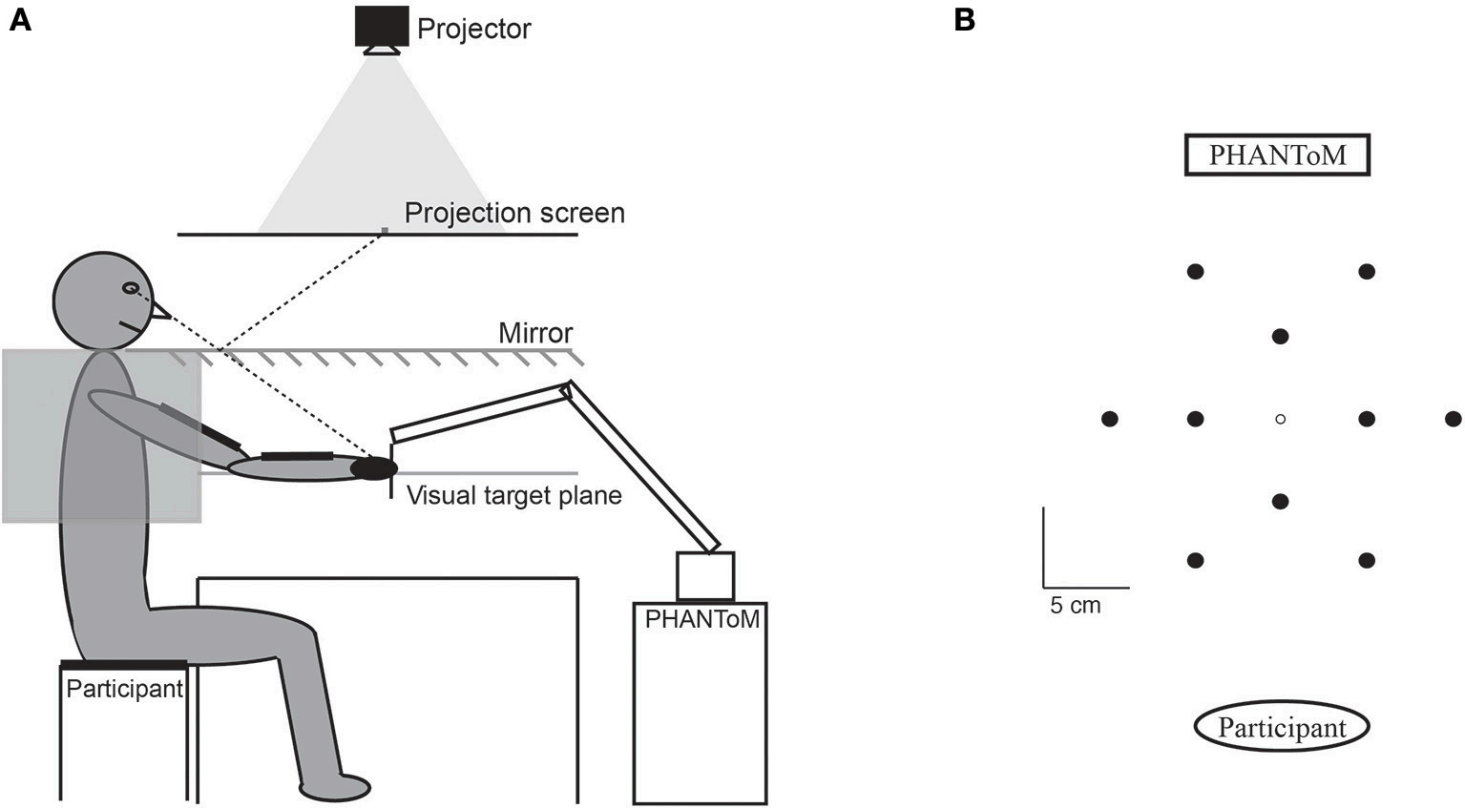
**Set-up. (A)** Experimental set-up. The subject could not see his hand, his arm, or the tape. **(B)** Top view of the virtual target positions (filled circles) relative to the origin (open circle) and the participant and PHANToM (both not to scale). At each moment only a single target position was visible.

We used the same set-up as in our previous experiment (Kuling et al., 2013). We projected the visual target stimuli (always only one target present) on a white rear-projection screen above a mirror. The mirror reflected the projected image, so that the subjects perceived the targets in a horizontal plane below the mirror (Figure 1A). Subjects moved their hand below the mirror, holding a PHANToM Premium 3.0/6DoF (SensAble Technologies) force feedback device that measured the position of the hand and could be used to create a force field. Subjects could never see their hand or arm and their wrist joint was fixated with a tight inline-skating wrist protector.

A visual target was shown at one of ten possible target positions arranged around an origin (open circle in Figure 1B), which was aligned with the midline of the subject's torso and about 30 cm in front of the subject. Six outer target positions were at a distance of 10 cm from the origin. Four inner target positions were at a distance of 5 cm from the origin. The color of the disc provided feedback about the height of the handle so that the subject would not hit the mirror. The target was green as long as the handle was not more than 3 cm above or below the plane of the targets. Above this range the target turned red, and below this range it turned blue. Subjects were informed about this color-coding.

In our previous experiment (Kuling et al., 2013), we found that force fields did not influence reaching in a visuo-proprioceptive matching task. Because proprioception is based on more information sources than just muscle spindles, this result could be explained by the fact that subjects had unperturbed cutaneous and joint information. Possibly something similar could happen when manipulating skin stretch; joint and muscle information could be sufficient to compensate for the manipulation of the skin. Therefore, we repeated our previously ineffective force field in combination with the skin stretch manipulation. We anticipated that the combination of the two manipulations would lead to larger effects on the reached position of the hand than the sum of the effects of the individual manipulations. The force field that was used was identical to the position-dependent clockwise force field we used before (Kuling et al., 2013). The forces were in the horizontal plane, perpendicular to the direction toward the origin (a central point in the workspace, aligned with the midline of the subject's torso and about 30 cm in front of the subject). The magnitude of the forces was zero at the origin and increased with the horizontal distance from the origin by 25 N/m. The forces were independent of the vertical position of the handle.

### Tape application

**Figure 2:**
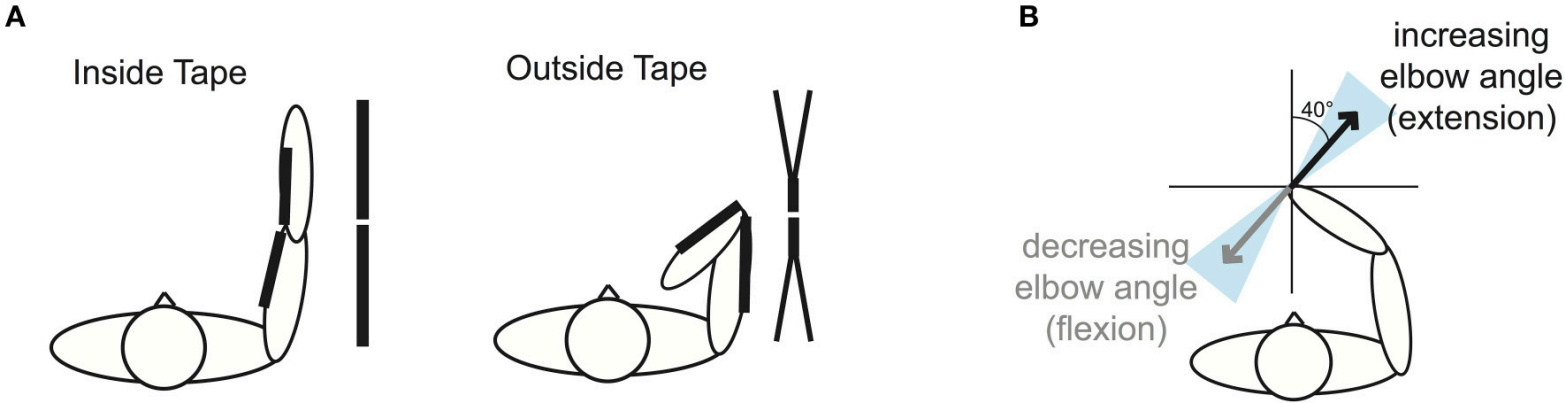
**Taping methods. (A)** Schematic drawing of the taping methods in experiment 1. The Inside Tape was a straight line of tape on the inside of the arm, applied in complete arm extension and with the tape fully stretched. The Outside Tape had a cross-form and was applied on the outside of the arm in complete arm flexion and with the tape fully stretched. Both tapes were cut at the elbow joint to prevent them from restricting the movements and in both sessions the tape did not cover the whole arm. **(B)** Illustration (not to scale) of the expected (range of) direction(s) of the translational shift in the reached position of the hand for changes in perceived elbow angle. The black arrow shows the mean direction of the expected shift, while the shaded area indicates the range of directions of the expected shift.

The taping of our subjects was inspired by examples of medical taping and was done in a way that would manipulate the skin stretch, but not restrict the movement of the elbow joint mechanically (see Supplementary Material for a video S1 of the tape application). New pieces of tape were used for each subject and session. The width of the unstretched tape was 5 cm. The total length of the unstretched tape that was used for each subject was the length of the subject's arm divided by two, –5 cm. The center of the tape was applied to the skin at the elbow joint and the tape was then cut at this position to prevent it from restricting the joint mechanically. In the *Inside Tape* session, the tape was then fully stretched and applied to the inside of the arm when the latter was in complete extension (Figure 2A, Inside Tape). In the *Outside Tape* session, the pieces of tape were partly split along the length of the tape, fully stretched, and then applied to the outside of the arm when the latter was in complete flexion, creating a kind of “cross” (Figure 2A, Outside Tape). The tape was cut in a cross-shape for no particular reason, other than because it is a common pattern in sports applications of the tape. In both the *Inside Tape* session and the *Outside Tape* session the tape did not cover the whole arm. Because of possible differences in skin stretch between subjects, the precise forces along the skin of the arm could not be perfectly regulated or even known precisely. Although the stretching of the skin is not precisely the same for all subjects, it should be similar in terms of direction and order of magnitude. Therefore, the length of the tape was sized according to limb length of each subject.

### Procedure

Before each session, the subjects received verbal instructions about the task. They had to move their dominant hand to the position of the target in one fluent movement. There were no time constraints, so subjects had ample time to correct any programming errors. When subjects thought that their hand was aligned with the target, they had to press the button on the handle of the force-feedback device. At that moment, the target disappeared and the next target appeared; subjects moved from target to target. Subjects did not receive any feedback during the experiment other than from their own proprioception. Previous studies have shown that there is no significant drift or accumulation of errors in this paradigm. van den Dobbelsteen et al. (2001) showed that reaching for visual targets is mainly based on matching the intended endpoint (endpoint coding), so that we expect the endpoints to be independent of previous errors. The results in our previous experiments with the same task as the current experiment (Kuling et al., 2013, 2015) indeed did not show an accumulation of errors (drift) over trials. The force-feedback device tracked the position of the subject's hand during the whole experiment. We consider the position at the moment of button-press to be the matched position.

Each combination of tape and force field was presented to every subject in blocks of 102 trials. The first and the last (102nd) trial of each block were toward a target located at the origin to avoid abrupt forces to the arm when switching the force field on and off. These two trials were not considered in the analysis. The 100 remaining trials consisted of 10 sequences that each contained the 10 target positions in random order (making sure that the first position of a sequence was never identical to the last position of the previous sequence).

Table 1 lists the five blocks of trials that were presented in the two sessions. The blocks were presented in a semi-counterbalanced order across subjects. The two blocks with tape were always preceded by the *Baseline* block and the *Force* block without tape, and followed by the *After* block. This last block was introduced to check whether removing the tape would induce some kind of aftereffect. The order of the two sessions and the order of the blocks with and without force were counterbalanced, resulting in 8 possible order combinations, which were each presented to two of our 16 subjects. The two sessions took place on different days within a 2-week period. A block of trials took the subject 4–7 min. After each block there was a break of 3–5 min. On average, it took the subjects 45 min to complete each session.

**Table 1:** **Experimental design of experiment 1**.

**Inside tape session**	**Outside tape session**
Baseline	Baseline
Force	Force
Tape	Tape
Tape + Force	Tape + Force
After	After

*Overview of the five blocks in each of two sessions in experiment 1. The order of the two sessions and the order of the blocks within each cell were counterbalanced across subjects*.

### Analysis

For each subject and block, the mean end point was calculated for each target position and compared with the position of the visually presented target to determine the position errors. We checked whether there was any drift in the position errors by comparing the mean matching error for each repetition (mean of all targets) with 10 × 4 repeated measures ANOVAs (repetition × block) per session.

Next, we used the differences between the position errors in the different blocks to search for any systematic effect of the manipulations. For three of the blocks (*Force, Tape*, and *After*) we defined error fields as the set of difference-vectors between the position errors in that block and those in the *Baseline* block of that session. As we were primarily interested in the effect of tape, we defined the error field for the *Tape* + *Force* block relative to the *Force* block of that session (this choice will not have substantial effects, as the force field alone has no significant effect on this task; Kuling et al., 2013).

Because the tape was applied on the arm around the elbow, we expected the tape to have an influence on the perceived elbow angle, which would correspond roughly to a translation perpendicular to the lower arm. We estimated the direction of this predicted effect by measuring the angles perpendicular to the lower arm for a typical subject at all target positions. The mean of these angles predicts that an increase in elbow angle corresponds with a translation of 40° (range of 25–55°; see Figure 2B). We expected the reduced stretching of the skin along the inner arm due to the tape to make it feel as if the elbow joint was more flexed. To overcome this, subjects would have to extend their elbow slightly more, which would result in a translation of the perceived positions in a direction of about 40°. For the tape on the outside of the arm we expect a translation in the opposite direction: A shift in end-points in the direction of more elbow flexion.

Based on these predictions, we examined how much of the error field could be explained by a uniform translation. To test whether there is a systematic influence of the force field (expected to occur in the *Tape* + *Force* block) we examined how much of the error field could be explained by a rotation around the origin. Both tests were also used in our previous experiments (Kuling et al., 2013, 2014).

For the *Force, Tape, Tape* + *Force* and *After* blocks, we determined the rotation and translation that described the error field best by minimizing the unexplained variance over all targets. This was done separately for each subject and session. The rotation was analyzed per session with a one-way repeated measures ANOVA with factor block (4 levels) on the rotation angle of the best fits. We analyzed the vector that best describes the translational error field (refered to as “translation” from now on) in a custom way. For each translation, we determined a 95% confidence ellipse of the mean of subjects' errors by dividing the axes of the 95% confidence ellipse for the distribution of the translations (of all subjects, calculated with the use of the covariance matrix) by the square root of the number of subjects. Because we want to compare the differences between blocks in two dimensions, it is not possible to use *t*-tests to compare the means. By calculating 95% confidence ellipses of the means we can easily see whether a difference is significant. If the origin lies outside this confidence interval it would be very unlikely (≤ 5% chance) that this point is part of the same distribution. In this way our analysis is similar to one-sample *t*-tests.

### Control experiment 1

In control experiment 1 we measured the skin stretch pattern on the inside and outside of the dominant arm during the kind of arm movement made in our task to get more insight in how the skin stretches during elbow movements.

#### Apparatus

To record the skin stretch along the length of the arm, Optotrak markers (infrared emitting diodes) were positioned on the inside or outside of the arm. In each case, they were positioned at the middle of the elbow joint and every 3 cm from this point along both the lower arm and the upper arm (**Figure 5**), resulting in 15–17 markers per arm, depending on the subject's arm length. The positions of the markers were recorded at 200 Hz during arm extension movements.

#### Procedure

Subjects held their arm at shoulder height with an elbow angle of ~90° and the hand in a fist. From this starting position, subjects extended their arm to full extension. After holding the arm in this position for a few seconds, subjects moved their extended arm to the side. This movement was first done with the markers on the inside of the arm and repeated with the markers on the outside of the arm.

#### Analysis

We analyzed the part of the movement between an elbow angle of 150 and 165°, because this is when the posture is similar to the movements made in Experiments 1, and represents a displacement of the hand of about 7 cm. The distances between consecutive markers were calculated for all markers on both sides of the arm. We looked at the change in these distances during 15° of elbow flexion to get some insight into the normal distribution of changes in skin stretch across the arm.

### Control experiment 2

This control experiment was designed to test whether increasing the amount of stretch on the tape leads to a larger effect on the position of the hand, and whether applying the tape differently would affect the results. To achieve the latter, we changed the cross-shape into a straight line of tape on the outside of the dominant arm (as was used on the inside of the arm in the main experiment) and attached the tape to the full length of the upper and lower arm.

#### Methods

We used the same set-up and task as in the main experiment. Three tape configurations were tested in separate sessions: Fully stretched tape on the inside of the arm (*Inside Tape full*), less stretched tape on the inside of the arm (*Inside Tape light*) and fully stretched tape on the outside of the arm (*Outside Tape full*). The tape on the outside of the arm was applied in the same manner as on the inside. We hypothesized that the direction of the translation in the *Inside Tape full* would be about 40° (range 25–55°), as in the main experiment. For the *Inside Tape light*, we expected a smaller translation than with the shorter tape, in the same direction. This would be in line with the findings of Collins et al. (2005) who showed that weak stretch on the skin evoked a smaller effect on proprioceptive posture matching of the two arms than larger stretch. Again, the tape in the *Outside Tape full* session was expected to give a translation in the opposite direction. The data was analyzed as in the main experiment.

#### Taping

The length of the unstretched tape was 66% of the combined length of the subject's upper and lower arm for the *Inside/Outside Tape full* session and 80% of the combined length for the *Inside Tape light* session. We chose 66% because the tape can be stretched to ~150% of its own length, and unlike in the main experiment we wanted the tape to cover the full length of the arm. The amount of force that was needed to stretch the tape from 66% of the arm length to the full arm length was about 4N. For the *Inside Tape light* session, the force that was needed to stretch the tape to the full arm length was about 2N. These forces are very rough estimates of the forces in the tape along the stretched direction, measured in pieces of tape that were not attached to the arm. The tape was applied in separate pieces for the upper and lower arm. Both pieces of tape started about 1 cm from the elbow joint in order not to restrict movements of the joint. They were always stretched to the other end of the body part. This procedure differed from that in the main experiment, in which we first applied the tape and afterwards cut it at the joint. This different procedure was used to get the same tape-free zone at the joint for all subjects and both sides of the arms.

#### Procedure

The three sessions with different tape configurations were measured on different days to avoid any aftereffects. Each session consisted of two blocks: A *Baseline* block and a *Tape* block. The blocks were always presented in that order. We compared the matched positions with and without tape within each session.

## Results

### Main experiment

The data of two subjects for the *Outside Tape* session were lost due to a computer problem. One subject did not show up for the second session so her data are missing in the *Inside Tape* session. Because we did not compare the results between sessions directly, we could use the results of the remaining session for these three subjects. Trials took about 1.5–2.0 s, indicating that subjects performed the task as a matching task as was instructed.

**Figure 3:**
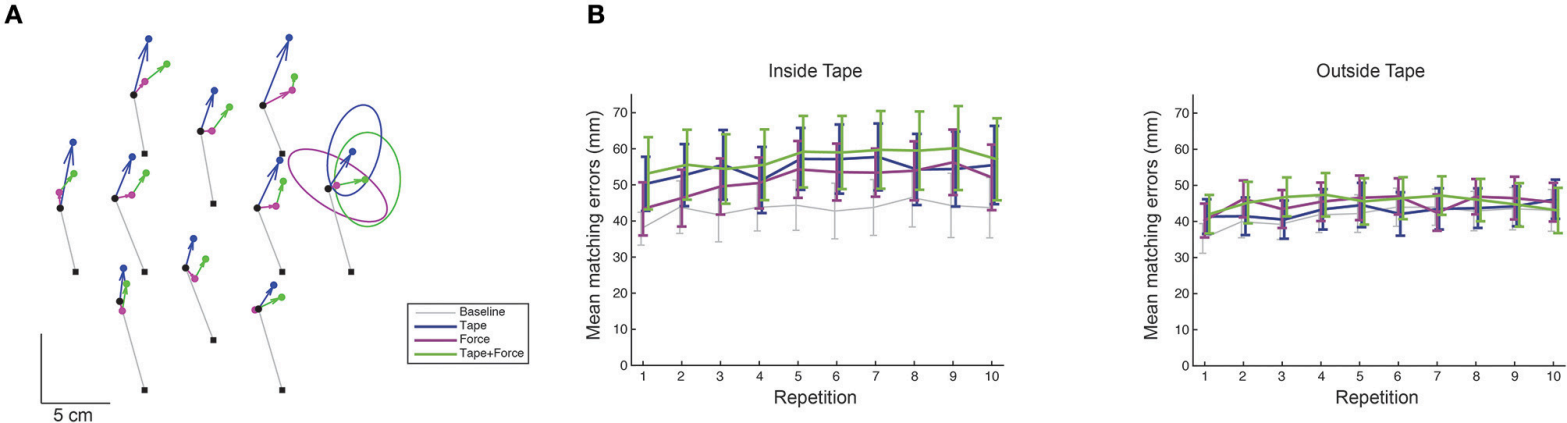
**Matching errors. (A)** Error fields of an example subject in the main experiment, session Inside Tape. The black squares are the target locations. The black dots are the end-points in the Baseline block. The thin gray lines indicate the errors in the Baseline block. The colored arrows show the error fields (the differences between the errors between blocks). For the Force and Tape, these are expressed relative to the baseline block; for the Tape + Force relative to the Force block. Note that the effects of the manipulations (the error-fields, colored lines) are much smaller than the error made in the baseline block (gray lines). For clarity, the results of the After block are not shown in this example. The blue and green arrows all point in similar directions, while the magenta ones are much more variable in their directions (and smaller). This suggests that the effect of tape is a uniform translation with and without force, whereas force itself has no systematic effect. Ellipses representing the 95% confidence intervals of the mean are shown for the rightmost target. **(B)** The mean matching errors for all repetitions. There is no significant drift during the experiment.

For each subject and session (15 subjects for the *Inside Tape* session and 14 subjects for the *Outside Tape* session), the mean perceived positions were calculated and compared with the visually presented target positions. Consistent with the subject-specific patterns of errors found in other experiments (Smeets et al., 2006; Sousa et al., 2010; Rincon-Gonzalez et al., 2011; Kuling et al., 2013, 2014, 2015; van der Kooij et al., 2013), the individual pattern of errors differed considerably between the subjects, but in general resembled a consistent translation for each subject (see data for an example subject in Figure 3A). The repeated measures ANOVAs for both the *Inside Tape* session and the *Outside Tape* session showed no significant effects of drift [*F*_(9, 20.7)_ = 2.95, *p* = 0.081, and *F*_(2.7, 34.5)_ = 2.59, *p* = 0.075 respectively, Figure 3B]. An effect of block on the magnitude of the matching error was only found in the *Inside Tape* session [*F*_(3, 36)_ = 4.44, *p* = 0.009, *Outside Tape* session: *F*_(1.8, 23.1)_ = 0.64, *p* = 0.517, Figure 3B]. There were no significant interactions. *Post-hoc* comparisons with Bonferroni correction show that in the *Inside Tape* session the mean matching error was significantly larger in the Tape block compared to the Baseline block (*p* < 0.05). There were no other significant comparisons.

All subjects' error fields (i.e., the effects of the manipulations) could be well approximated by a translation: The translations explained between 65 and 75% of the errors. The rotations could only explain between 8 and 10% of the errors independent of whether there were force fields or not. The repeated measures ANOVAs for both the *Inside Tape* session and the *Outside Tape* session showed no differences between the best fits for all blocks [*F*_(3, 42)_ = 1.21, *p* = 0.317, and *F*_(1.3, 17.4)_ = 0.18, *p* = 0.749 respectively], which suggests that the rotations were not systematically related to the direction of the force field.

**Figure 4:**
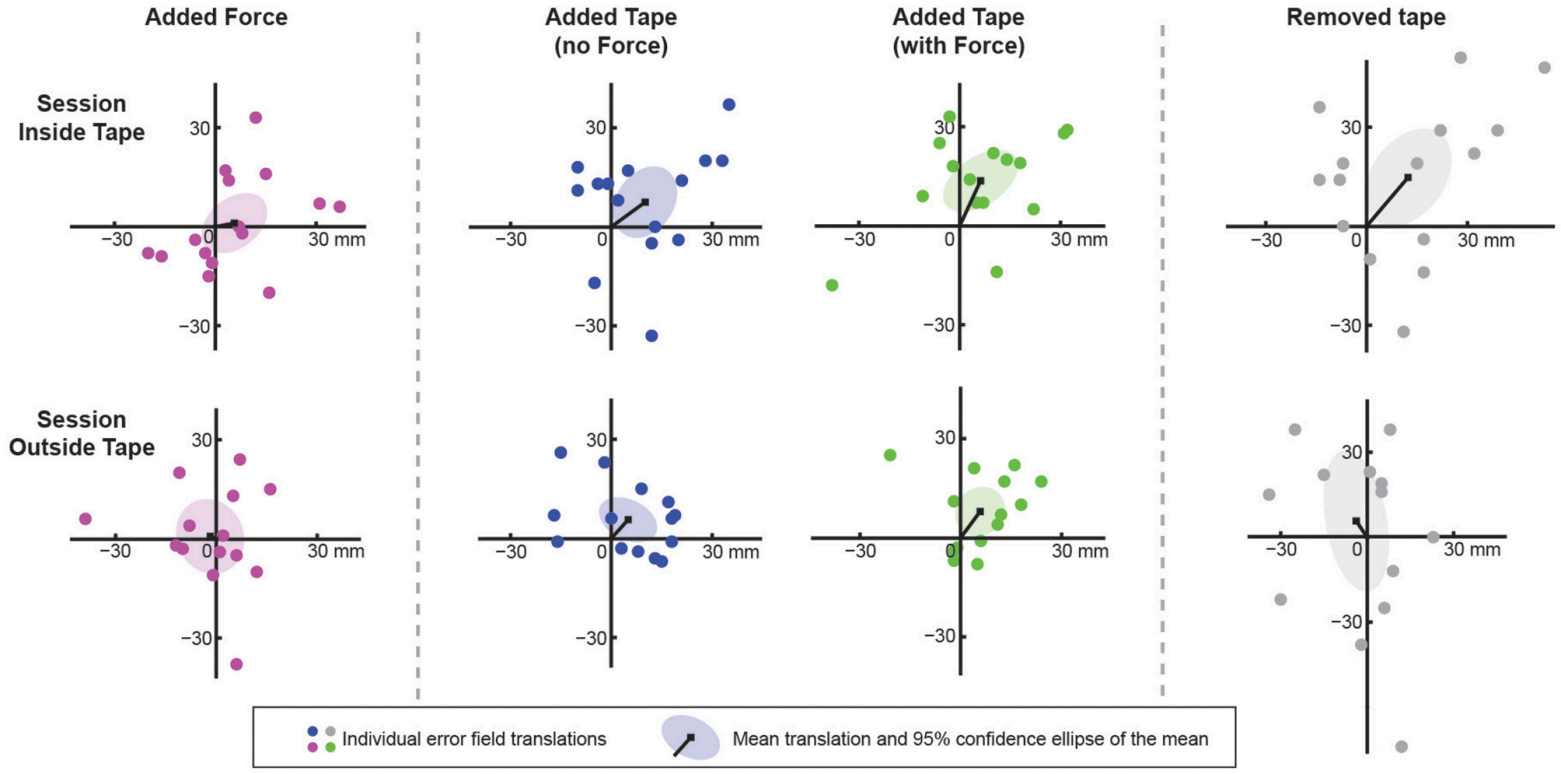
**Results of the main experiment**. Each dot indicates the fitted translation (scales in mm) for an individual subject's error field. The black lines to the black squares show the mean translation. The ellipses show the 95% confidence intervals of the mean translation caused by the manipulation in question. The two panels on the left show that added force did not result in a significant translation. The four central panels show that added tape resulted in a significant translation in the direction of elbow extension for both taping methods. The two panels on the right show that the consistent translation caused by the Inside Tape remained present after removal of the tape, while the translation caused by the Outside Tape did not (both compared with the Baseline block). The between-subject variability in the effect of removing the tape is much larger than that in the effect of other manipulations.

In Figure 4, the best fitting translations are shown for all subjects (colored dots) in both the *Inside Tape* session and the *Outside Tape* session. A point at the origin would indicate that the two blocks that are compared yield the same error (i.e., the manipulation has no significant effect). Dots indicate the translations for individual subjects relative to the *Baseline* block (except for the *Tape* + *Force* blocks which are plotted relative to the *Force* blocks). The ellipses indicate 95% confidence ellipses of the means. As expected, the origin lies within the ellipse for the *Force* block in both sessions, which shows that there is no systematic effect of the presented external force on the perceived proprioceptive position sense of the hand (confirming the results of Kuling et al., 2013).

A systematic translation can be seen in all four blocks involving tape (the origin lies outside the 95% confidence ellipses of the means). Note that the translation values for the *Tape* + *Force* blocks represent the effect of the tape, because these are plotted relative to the *Force* block. The directions of the mean translations of the *Inside Tape* and *Inside Tape* + *Force* are 53° and 39°, which are within the expected range. For the *Outside Tape* and *Outside Tape* + *Force* blocks a similar systematic effect can be seen; the directions of the mean translations are 42° and 24°, respectively, which is opposite to the expected direction.

In the *After* blocks, the variability in the translations between subjects is much higher than for the other blocks (compare the size of the ellipses in the panels on the right of Figure 4 with the size of the other ellipses). For the *After* block in the *Inside Tape* session, the translation is in the same direction (40°) as the translation in the *Tape* blocks, while for the *Outside Tape* session no systematic effect was found. The results in the *Baseline* blocks did not differ systematically between the two sessions (not shown), so the distorting aftereffects of the tape of the first day did not transfer to the second day.

### Control experiment 1

**Figure 5:**
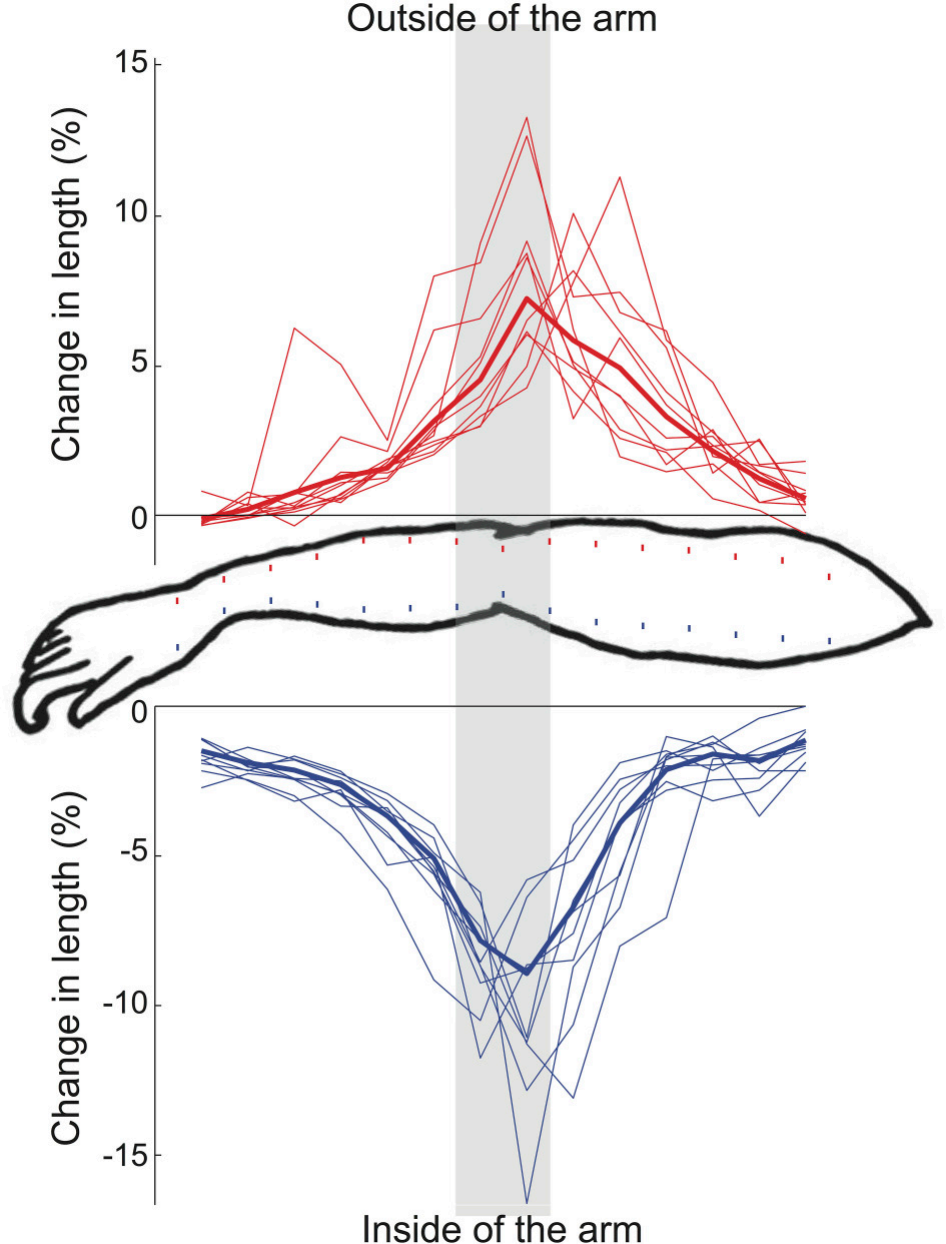
**Results of control experiment 1**. Change in skin stretch corresponding to an elbow flexion movement from 165 to 150° for both the inside of the arm (blue) and the outside of the arm (red). Each thin line represents the data of one subject; the thick lines show the mean over 11 subjects. The dots on the arm drawing give a schematic representation (not to scale) of the marker placement. The distances between consecutive markers are used as a measure of skin length and compared between the two elbow angles. The difference between these lengths, as a percentage of the lengths themselves, provides a measure of local skin stretch.

The stretch of the skin due to 15° elbow flexion was similar for all subjects (Figure 5). On both the inside and outside of the arm, the skin stretch changed the most at the elbow joint (about 9% and 7%, respectively for the 15° movement). The changes gradually decreased toward the proximal and distal ends of the arm (Figure 5), again in much the same way for the skin on both sides of the arm.

### Control experiment 2

**Figure 6:**
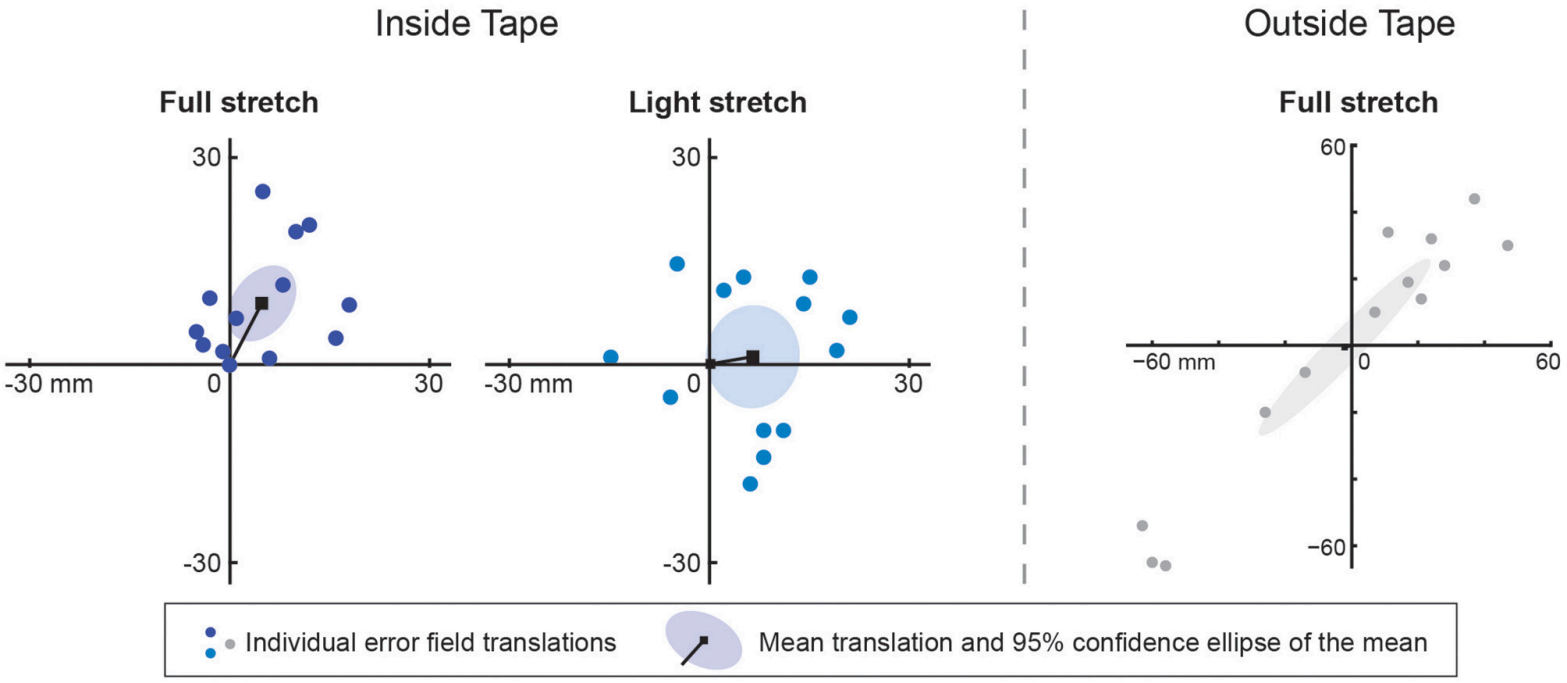
**Results of control experiment 2**. Each dot indicates the fitted translation (scales in mm) for an individual subject's error field. The black lines to the black squares show the mean translation caused by the manipulation in question. The ellipses show the 95% confidence intervals of the mean translation. There was a systematic translation in the expected direction for the Inside Tape full session (left panel), but not for the other two sessions. Note the difference in scale of the axes between the inside and the outside tape sessions.

The fitted translations for all subjects in each of the three sessions can be seen in Figure 6. For the *Inside Tape full* session the patterns were translated 28° clockwise from the body's midline, which is within the expected range of directions of 25°–55°, confirming the results for *Inside Tape* in the first experiment. For the *Inside Tape light* session, a tendency can be seen in about the expected direction, but the translation is not significant. There is a lot of variability between subjects. We expected the individual translation vectors to be smaller in this block compared to those in the *Inside Tape full* session, but this was not so. The results of the *Outside Tape full* session show no significant translation. There are large differences between the subjects in the effect of applying tape to the outside of the arm. In this session, all translations seem to be along the expected axis, but for 8 subjects (dots in the upper right quadrant) the elbow was extended more than without tape, while the other 5 subjects flexed their arm more, as expected (dots in the lower left quadrant). The effects for the individual subjects are much larger than in the two other sessions: For five subjects, the tape caused more than 5 cm displacement, whereas in the other two sessions, all individual translations were less than half of this.

## Discussion

In this study, we applied elastic tape to the inside or outside of the arm to manipulate the skin stretch patterns around the elbow, and examined the effect of doing so with a visuo-proprioceptive matching task. When matching the unseen hand to a visual target, systematic subject-specific errors were found. This is consistent with the subject-specific patterns of errors found in other experiments (Smeets et al., 2006; Sousa et al., 2010; Rincon-Gonzalez et al., 2011; Kuling et al., 2013, 2014, 2015; van der Kooij et al., 2013). The errors were of the same order of magnitude (several centimeters) as was found in previous studies with a similar set-up (Kuling et al., 2013, 2014, 2015). We have previously shown that these errors are stable across long periods of time (many weeks; Kuling et al., 2016). These initial errors themselves were not further analyzed in this study. Each subject's error without attached tape was used as a baseline measure for their performance with the skin stretch manipulations. The baseline was measured either without or with forces (although forces have been shown not to influence the initial errors, Kuling et al., 2013) in order to isolate the effect of the skin stretch manipulations.

In the main experiment, we used elastic tape to manipulate the skin stretch patterns around the elbow. In the *Inside Tape* session, the stretched tape was applied on the inside of the arm. We expected a translation of the perceived positions in a direction of about 40° (more elbow extension). The results of the *Inside Tape* session indeed show a translation in this direction. The effect of tape was independent of the force on the arm.

In the *Outside Tape* session a translation in the opposite direction was expected, because the tape was on the opposite side of the arm. However, we found a translation in almost the same direction as in the *Inside Tape* session. Thus, although changing the natural skin stretch with elastic tape clearly induced systematic changes in elbow angle when people had to rely on proprioception for localization, the relationship between skin stretch and judged posture was not straightforward because we found an asymmetry between applying tape on the inside and outside of the arm.

What could explain this asymmetric effect of the tape? An assumption underlying the expectation that tape would have opposite effects on opposite sides of the arm is that the skin stretch differences during a displacement are opposite, but similarly distributed across the arm. In control experiment 1 we found that the changes in the skin stretch during the movement are distributed in a very similar way across the arm (although the skin is obviously stretched when moving in the opposite direction) for the inside and the outside of the arm. Therefore, the asymmetry found in the main experiment cannot easily be explained by differences in the relation between natural skin stretch patterns and joint angle.

Another explanation for the asymmetric effect in the main experiment might be that the method we used to put the tape on the outside of the arm (the cross-form) was not exactly the converse of the tape on the inside of the arm (straight lines). In control experiment 2, we reproduced and extended the results found in the main experiment for the tape on the inside of the arm with a new group of subjects, showing that the translation of the matched hand position due to tape on the inside of the arm is quite robust. The results of the tape on the outside of the arm were different from the results in the main experiment, but again not as expected. Some of the subjects showed a clear translation in the expected direction, while others showed a clear effect in the opposite direction (as in the main experiment). In this experiment we also tested whether the amount of skin stretch has an influence on the magnitude of the effect. The differences between the full stretch tape and the light stretch tape show that indeed a certain amount of stretch is necessary to get a significant shift in reached endpoints. This rules out the option that attaching anything to the subject's arm induces a change in posture.

In the literature about proprioception, skin stretch information is considered to be less important than muscle information. However, in our study we showed that the manipulation of skin stretch induces a change in perceived position of the hand, although there was normal afferent information from the muscle spindles and joint receptors in the trials with the tape applied, while force manipulations did not show a systematic change in the same task. Moreover, the effect of the tape did not differ substantially when force was added, so it was presumably due to the sensory consequences of stretching the skin rather than to the modest forces resulting from it. This supports earlier findings that suggest that skin stretch is an important factor in proprioceptive localization (Edin and Abbs, 1991; Edin, 1992, 2001; Edin and Johansson, 1995; Collins and Prochazka, 1996; Collins et al., 2000, 2005) and suggest that the influence of information about skin stretch might have been underestimated in comparison with muscle information in the past.

In our experiment, we applied tape to introduce skin stretch. The tape does so by introducing forces parallel to the skin. If the tape would connect pieces of skin on both sides of the elbow joint, these parallel forces would directly lead to torques around that joint. However, the tape was cut at the joints to ensure that this would not happen. In principle, the skin stretch patterns we applied may lead to torques around joints due the connective tissue. So the effects of skin-stretch might not be due to effects beyond the perceptual effect of sensors in the skin.

Whereas the tape on the inside of the arm produced a robust translation in the matched target position, the tape on the outside of the arm did not result in a clear reproducible translation. One possible factor influencing the differences between tape on the inside and the outside of the arm could be sensitivity differences between the inside and the outside of the arm. From the literature we know that skin characteristics (i.e., hairiness, sensitivity for point localization, and two-point thresholds) differ *between* body parts (Lederman and Klatzky, 2009), but it is unclear whether and how the sensitivity exactly varies *within* body parts. Studies using the two-point threshold method have shown that there are differences in sensitivity for lateral, medial and posterior parts of the upper, and lower arm (e.g., Nolan, 1982; Shibin and Samuel, 2013). In general, the lower arm is more sensitive than the upper arm. Within the upper arm there are large differences between the upper parts (less sensitive) and the lower parts (more sensitive). In both the upper arm and the lower arm the lateral parts are less sensitive than the medial and posterior parts. Because the tape was not strictly applied within the described areas it is hard to directly relate the sensitivity of the arm to our results. However, it might be an explanation why the effects of the tape application on the outside of the arm were different between experiment 1 (tape in cross-form, on posterior, and lateral parts) and control experiment 2 (tape mainly on the posterior parts), and it might also explain why the similar taping on the inside and outside of the arm did not show clear opposite effects. The asymmetry could of course also be caused by asymmetries at other levels, for instance the connectivity between skin and underlying muscle tissue (e.g., Chaudhry et al., 2014).

An interesting question related to the source of the asymmetry is which skin stretch patterns give the most important proprioceptive information. Is it the large area of skin to which the tape is applied, as we have hitherto assumed, or the small area of skin between the two parts of tape (which is the skin over the joint)? Applying tape on the arm does not only result in reduced stretching along the inner/outer arm, but also in increased stretching of the skin on the elbow joint. The skin on the elbow joint is more stretched because of the tape and to overcome this, one might expect more flexion with tape on the inside of the arm and more extension with tape on the outside of the arm. So the direction of the predicted effect depends on which part of the skin contributes most to the percept. This might differ between the skin on the inside and the outside of the arm, and even between subjects. Further research is needed to fully understand the influence of skin stretch patterns on the arm. However, the present study demonstrates that skin stretch plays an important role in knowing the position of the hand.

From the present explorative study we can also conclude that elastic tape can be used for non-invasive research on the contribution of cutaneous receptors in proprioception. Manipulating the skin stretch of the arm with tape changed proprioceptive localization in our visuo-proprioceptive task. With tape on the inside of the arm, the direction of the proprioceptive shift was quite robust following our initial predictions. With the tape on the outside of the arm the results were more variable, suggesting that the influence of cutaneous receptors on proprioception is more complex than we had anticipated when making our predictions. By comparing different ways of taping one may be able to unravel some of this complexity.

## Author contributions

Conceived and designed the experiments (IK, EB, JS). Performed the experiments (IK). Data analysis (IK, EB, JS). Wrote the paper (IK, EB, JS).

### Conflict of interest statement

The authors declare that the research was conducted in the absence of any commercial or financial relationships that could be construed as a potential conflict of interest.

## Supplementary material

Video S1 **Application of the tape**.
